# Outcomes in patients with chronic lymphocytic leukemia and *TP53* aberration who received first-line ibrutinib: a nationwide registry study from the Italian Medicines Agency

**DOI:** 10.1038/s41408-023-00865-z

**Published:** 2023-06-28

**Authors:** Gian Matteo Rigolin, Pier Paolo Olimpieri, Valentina Summa, Simone Celant, Lydia Scarfò, Lucia Tognolo, Maria Pia Ballardini, Antonio Urso, Mariarosaria Sessa, Silvia Gambara, Francesca Cura, Monica Fortini, Paolo Ghia, Antonio Cuneo, Pierluigi Russo

**Affiliations:** 1 Hematology unit, Department of Medical Sciences, University of Ferrara, Ferrara, Italy; 2 Italian Medicines Agency, Rome, Italy; 3 Università Vita-Salute San Raffaele and IRCCS Ospedale San Raffaele, Milan, Italy

**Keywords:** Chronic lymphocytic leukaemia, Targeted therapies

## Abstract

In this analysis we describe the effectiveness of first-line ibrutinib in 747 patients with chronic lymphocytic leukemia (CLL) and *TP53* aberrations in a nationwide study with a 100% capture of patients who received the study drug. Median age was 71 years (range 32–95). An estimated treatment persistence rate of 63.4% (95% CI 60.0%-67.0%) and survival rate of 82.6% (95% CI 79.9–85.4%) were recorded at 24 months. Disease progression or death were the reasons for discontinuation in 182/397 patients (45.8%). A higher risk of treatment discontinuation was found to be associated with age, ECOG-PS and pre-existing heart disease, whereas ECOG ≥ 1, age ≥ 70 years and male sex were associated with an increased risk of death. Median post-progression overall survival (OS) was 12.2 months (95% CI 9.2–22.0). Post-discontinuation median OS in patients who discontinued ibrutinib for other reasons was not reached (95% CI 42.3 months – NA). Ibrutinib was an effective first-line treatment for CLL and *TP53* aberrations in patients treated at large academic centers and community practice hospitals. Clinical characteristics at baseline may influence the effectiveness of ibrutinib, whereas the experience of prescribing centers and multi-hit or single-hit *TP53* aberrations had no impact on outcome in this high-risk population.

## Introduction

The Bruton tyrosine kinase inhibitor (BTKi) ibrutinib was shown to achieve high overall response rate and prolonged disease control in chronic lymphocytic leukemia (CLL) [1, 2]. International guidelines [3–6] support ibrutinib treatment in patients with CLL experiencing disease progression as per iwCLL criteria [7], both in first line and in the relapsed/refractory setting.

However, the efficacy of ibrutinib in previously untreated high-risk CLL carrying 17p- and/or *TP53* mutations, here referred to as *TP53* aberrations, has been assessed in a limited number of patients enrolled in clinical trials [8, 9].

Considering that clinical trials have restrictive eligibility criteria at enrollment, the patient population may not entirely reflect the one encountered in the clinic and particular subgroups might be under-represented, making Real World Evidence (RWE) an important research tool to obtain relevant clinical information in addition to interventional studies [10–15]. In a recent real-world evidence (RWE) analysis of patients with CLL who had received first-line treatment with ibrutinib, shorter overall survival (OS) and time to treatment discontinuation (TTD) were documented in patients carrying 17p deletion [16].

The Gruppo Italiano Malattie Ematologiche dell’Adulto (GIMEMA) Working Party (WP) on chronic lymphoproliferative disorders established a collaboration with the Italian Medicines Agency (AIFA) to analyze the effectiveness of targeted agents in patients with CLL included in the AIFA drug registry, which covers all patients treated with ibrutinib within the drug reimbursement framework of Italian National Health Service (INHSe).

In this analysis, we present and discuss the results in terms of effectiveness of first-line ibrutinib in a large and unbiased cohort of patients with CLL carrying *TP53* aberrations.

## Methods

This analysis is based on data collected from the AIFA web platform of Monitoring Registries (wMRs), an administrative database whose main scope is monitoring the appropriateness of drug prescription in Italy [17]. Inclusion in the wMRS is mandatory to prescribe ibrutinib in the approved indications for CLL within the drug reimbursement framework of the Italian INHSe and there are no other channels through which patients can obtain commercial ibrutinib in the framework of INHS reimbursement. Therefore, all patients who received ibrutinib in the INHSe are evaluated prior the start of therapy and then followed during the entire treatment. Data collected in the wMRS included: (i) demographic and salient clinical data, (ii) drug prescription (one prescription might cover from 30 up to 90 days), (iii) response to treatment (response assessment was requested every 90 days), (iv) end of treatment form (date and reason for discontinuation) and, v) patient status (alive/dead). According to Italian regulations, monitoring of these parameters does not require any consent form or formal approval from ethical committees. Patients included in the registry received, however, information about the purposes of the monitoring.

The follow-up for treated subjects was calculated as the interval between the first and last drug administration for all patients, including censored patients.

The main outcomes of the study were the time to treatment discontinuation (TTD) and overall survival (OS). Other outcomes were best response in terms of overall response rate (ORR) and time to progression, death or intolerable toxicity leading to discontinuation (PDT). TTD was defined as the time occurring between the date of the first administration of ibrutinib and the date of treatment discontinuation for any cause, including death or lost to follow-up, plus half the days of medication covered by one prescription (see supplementary material). PDT was defined as the time occurring between the date of the first administration of ibrutinib and the date of treatment discontinuation for progression, death or toxicity, plus half the days of medication covered by one prescription. The patients who discontinued ibrutinib for reasons other than progression, death or toxicity were censored at the time of discontinuation. The average daily dose was calculated dividing the overall milligrams dispensed to each patient to the time to treatment discontinuation, this time considering the entire period covered by the prescription (30, 60 or 90 days). Being a theoretical valorization based on real-world data, average daily dose is affected by both momentary treatment interruptions (that will decrease the calculated average daily dose) and anticipated drug dispensation (increase the calculated average daily dose). Best response was calculated for each patient considering all evaluations performed during the first 16 months of treatment. This threshold was selected to account for minimum potential follow-up available to all patients of 16 months.

OS was defined as the time between the first prescription of ibrutinib and date of death; the death dates of patients included in the registry were obtained from the National Register Office for the resident population (ANPR), which is a central database maintained by the Ministry of the Interior of Italy (decree 82/2005, art. 62). Time-to-event analyses were performed according to the Kaplan–Meier method. The impact of the different covariates on TTD was evaluated using the Cox proportional-hazards model. In this case, proportional assumptions have been checked testing for independence between Schoenfeld residuals and time and by graphical inspection. Variables included in the model were selected using a forward approach. Mixed effects were evaluated using the coxme package based on Ripatti and coworkers [18] with two levels: Regions and centers. The likelihood-ratio test was used to select between fixed and mixed effects Cox models.

The association between baseline characteristics and outcomes was also assessed using an accelerated failure time (AFT) model, with an exponential parametric specification.

The impact of 17p deletion or *TP53* mutation alone or in combination in the same patients was analyzed only when available. A sensitivity analysis was performed by mean of a logistic regression, comparing all baseline characteristics between patients with or without a complete set of information on 17p and *TP53*. The presence of one of the 2 aberrations alone or in combination were evaluated by mean of weighted Kaplan-Meier plots. Weights were obtained using average treatment effect (ATE) estimand and a gradient boosting algorithm [19] with mutation status as class label and all other available baseline characteristics as attributes. The twang [20], survey [21] and jskm packages [22] were used to predict the weights (twang) and estimate and plot the weighted Kaplan–Meier (survey and jskm). Log-rank test was used to compare the survival distributions of single and multi-hit aberrations (*p* < 0.05 is considered significant).

All statistical analysis were performed using R, version 4.0.5 [23]. Figures were produced using the ggplot2 package [24]. Numerical variables were described using median with first and third quartile (q1-q3) values, categorical variables were described using frequencies.

## Results

Seven-hundred forty-seven patients with CLL and *TP53* aberrations treated in first line with ibrutinib between January 2016 and December 2020 in 157 hematology centers were included in this analysis. One-hundred patients were previously reported [25]. Data cut-off was set to May 2022; therefore, all patients have a minimum potential follow-up period of at least 16 months. The median follow-up (FU) was 26.0 months (IQR 13.8-40.6), the median FU for patients without events was 35.1 months (IQR 25.4-48.6).

The baseline characteristics are shown in Table 1. The median time from diagnosis was 11 months; median age was 71 years (range 32–95 years); 93.2% of the cases had ECOG performance status (PS) 0–1. Rai stages 0-II and III–IV were reported in 54.9% and in 45.1% of the patients, respectively; previous atrial fibrillation in 3.4%; lymph node >5 cm and/or severe splenomegaly and/or lymphocyte >25 × 10^9^/L in 73.1%. Renal impairment was reported in 9.1% of the patients and concomitant use of systemic anticoagulants in 4.0% of the patients.

**Table 1:** Baseline characteristics.

Variable	Total *N* = 747 (%)
Sex M/F	456 (61.0)/291 (39.0)
Age	
median	71 years (range 32–95)
<65/65–69/≥70 years	215 (28.8)/132 (17.7)/400 (53.5)
Median time from diagnosis months (IQ range)	11 (2–39)
ECOG performance status 0/1/≥2	421 (56.4) / 275 (36.8)/51 (6.8)
Rai stage 0/I/II/III/IV	45 (6.0)/159 (21.3)/206 (27.6)/195 (26.1)/142 (19.0)
Bulky and/or elevated lymphocytosis and/or severe splenomegaly yes/no	546 (73.1)/201 (26.9)
del 17p absent/present/not available	134 (18.0)/568 (76.0)/45 (6.0)
TP53 WT/mutated /not available	112 (15.0)/429 (57.4)/206 (27.6)
del 17p only / TP53 mut only /del 17p & TP53 mut^a^	112 (15.0)/134 (17.9)/250 (33.4)
History of atrial fibrillation yes/no	25 (3.4)/722 (96.6)
Pre-existing severe heart disease yes/no	21 (2.8)/726 (97.2)
Renal impairment (Creatine clearance < 70 ml/min) yes/no	68 (9.1)/679 (90.9)
Concomitant use of anticoagulant yes/no	30 (4.0)/717 (96.0)
Number of experienced/less experienced centers^b^	24 (15.3)/133 (84.7)
Patients treated at experienced/less experienced centers	312 (41.8)/435 (58.2)

^a^206 patients have reported del(17)p with no information on the presence/absence of TP53; 46 patients have TP53 with no cytogenetic analysis performed/reported. These patients are not included in the reported frequencies.

^b^Centers with at least 2 years of experience in the wMRS.

The best response at 16 months in terms of ORR was 77.7% of patients (CR 16.3%, 122/747; PR 61.4%, 459/747 patients). Response rates at different time points up to 16 months are reported in Supplementary Fig. 1. Between 12 and 16 months (corresponding generally to the fourth mandatory patient evaluation), ORR was 53.0%.

**Fig. 1:**
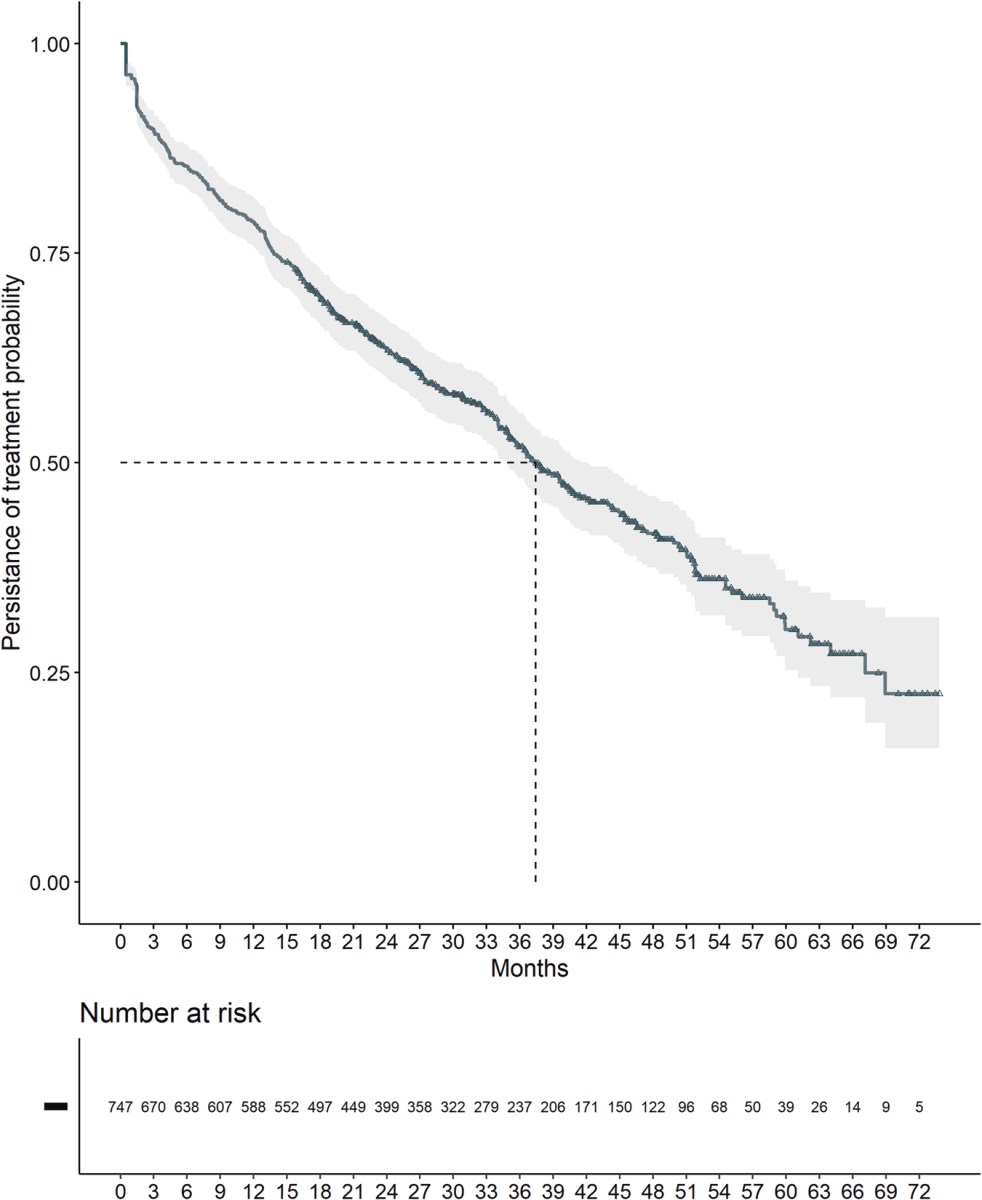
Time to treatment discontinuation (TTD). At the data cut-off (May 2022), 350 patients (46.9%) were still on ibrutinib and discontinuation occurred in 397 patients (53.1%) with a median time to discontinuation of 37.4 months and an estimated treatment persistence probability of 63.4% at 24 months.

At the data cut-off (May 2022) 350 patients (46.9%) were still on ibrutinib and discontinuation occurred in 397 patients (53.1%), with a median TTD of 37.4 months [95% CI: 34.8–42.2 months] (Fig. 1) and an estimated treatment persistence probability of 63.4% at 24 months [95% CI: 60.0–67.0%]. Disease progression (n. 108 patients) or death (n. 74 patients) were the reasons for discontinuation in 182/397 patients (45.8%), while 215/397 (54.2%) patients discontinued treatment for reasons other than progression or death including 108 patients who were lost to follow-up (Table 2). An advanced age and a higher Rai stage were associated with a higher risk of being lost while having a bulky disease with a lower risk (Supplementary Table 1). The median dose prescribed to these patients was 302,400 mg (2160 140 mg capsule). The most common daily dose adopted over the entire treatment was 420 mg was and less than 15% of the overall population had an average daily dose less than 300 mg (Supplementary Fig. 1 and Supplementary Table 2). At multivariable analysis a significantly higher risk of treatment discontinuation was found to be associated with age, ECOG-PS, history of atrial fibrillation and pre-existing severe heart disease (Table 3). Noteworthy, a slight deviation from the proportional assumption was observed for the ECOG class (*p* = 0.014). Even if the deviation was deemed not relevant upon graphical inspection of the fit of beta coefficient as a function of time, being largely restricted to the first and last days of follow-up (see Supplementary materials), we used an AFT model to back-up our findings, obtaining similar results. Patients with ECOG 2 or more showed a duration of treatment 51% shorter than patients with ECOG 0, confirming the association observed in the COX model.

**Table 2:** Reasons for discontinuation of ibrutinib treatment in 747 patients.

	N. of patients	%
**Discontinued**	397	100%
Progression or death	182	45.8
Discontinued except progression	215	54.2
Toxicity	37	9.3
Medical or patient decision	33	8.3
Lost to follow-up	108	27.3
Others (no drug related)	37	9.3

**Table 3:** Multivariate analysis for TTD and OS.

	HR (95% CI)
TTD variable	
Age years <65/65–69/≥70	
65–69 vs <65 years	1.16 (0.83–1.61)
≥70 vs <65 years	1.82 (1.41–2.35)
ECOG performance status 0/1/≥2	
1 vs 0	1.32 (1.07–1.63)
2+ vs 1	1.94 (1.35–2.77)
History of atrial fibrillation yes/no	1.80 (1.14–2.85)
Pre-existing severe heart disease yes/no	1.69 (1.03–2.77)
Renal impairment^a^ no vs yes	0.72 (0.52–0.99)
OS variable	
Gender M vs F	1.38 (1.01–1.87)
Age years <65/65–69/>70	
65-69 vs <65 years	1.30 (0.81–2.09)
≥70 vs <65 years	1.68 (1.15–2.45)
ECOG performance status 0/1/≥2	
1 vs 0	1.43 (1.05–1.95)
2+ vs 1	2.59 (1.63–4.12)
Pre-existing severe heart disease yes/no	1.77 (0.89–3.50)
Renal impairment^a^ no vs yes	0.72 (0.46–1.13)

^a^Creatine clearance < 70 ml/min.

**Fig. 2:**
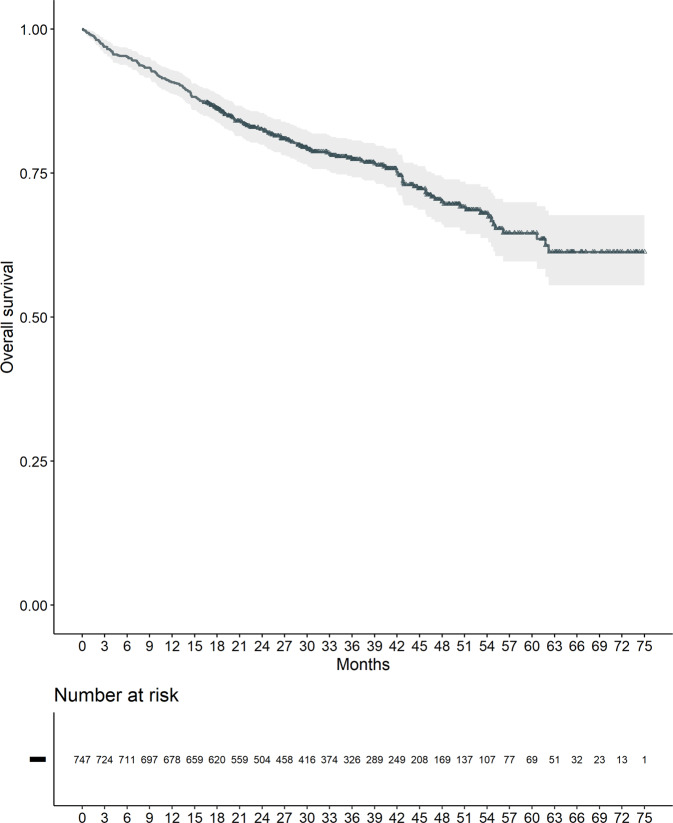
Overall survival (OS). Median OS was not reached with a 24-month survival probability of 82.6%.

Median OS was not reached, with a 24 months survival probability of 82.6% [95% CI: 79.9–85.4%] (Fig. 2).

As shown in Table 3, multivariable analysis showed that ECOG ≥ 1, age ≥70 years and male sex were significantly associated with an increased risk of death. No deviation in the proportional hazard assumption was found (Table 3). Interestingly, adding random effects to model the Italian health system as a two-level structure, did not significantly modify the results obtained by corresponding fixed effects models neither for TTD nor for OS (log-rank test *p* value = 1.00 and 0.74 for TTD and OS, respectively).

Median PDT was 62.2 months (95% CI: 55.1-NA), with a 76.6% [95% CI: 73.5–79.9] estimated PFS rate at 24 months.

**Fig. 3:**
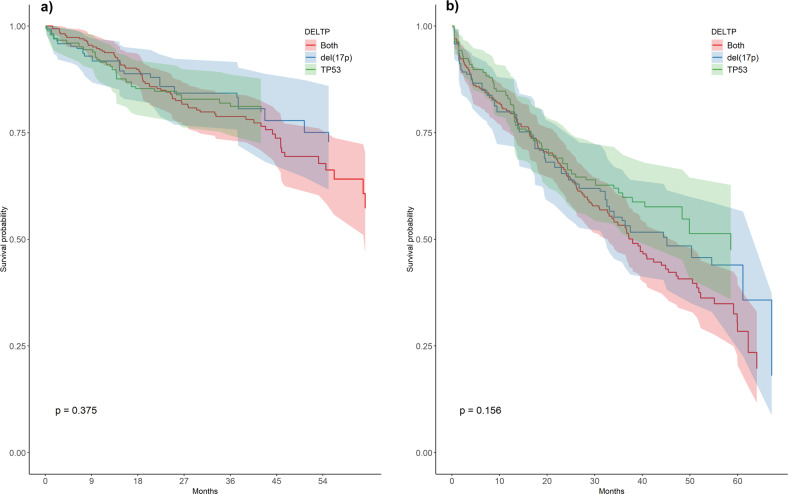
TTD and OS by single-hit and multi-hit *TP53* aberrations. In a subgroup of 496 patients (66.4% of the total population), *TP53* aberrations in combination in 250 patients had no significant impact on TTD (**a**) and OS (**b**) as compared with *TP53* deletion in 112 patients or *TP53* mutations alone in 134 patients.

The effect of coexistence of 17p- and *TP53* mutations compared to either del(17p) or *TP53* mutations alone was evaluated on the subgroup of patients for which information on both del/17p) and *TP53* were reported (*n* = 496 patients; 66.4% of the overall population). First, we looked for possible differences in the baseline characteristics among patients with and without the complete mutational status. A logistic regression was performed as a sensitivity analysis showing no significant differences that can point to non-random missingness in the reported data (see supplementary materials). Then, we built a gradient boosting algorithm to predict propensity scores and used them as weights in the Kaplan-Meier estimation of survival probabilities in order to balance the confounding effect of baseline characteristics. After adjusting, no baseline variables were different between the 3 subgroups (supplementary Fig. 5). Moreover, no significant differences were observed in weighted survival distribution of the three subgroups (Fig. 3) using log-rank tests (*p* = 0.374 and 0.156 for OS and TTD, respectively).

**Fig. 4:**
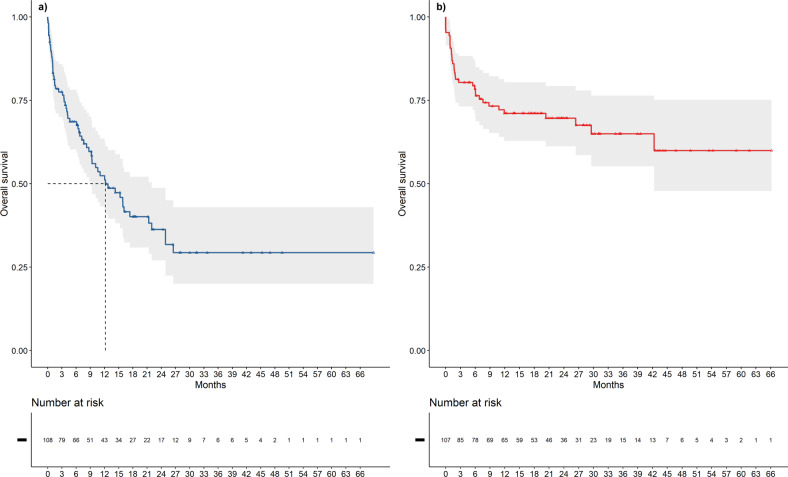
Post-progression OS and post-discontinuation OS. In 108 patients, median post-progression OS was 12.2 months (**a)**, while post-discontinuation OS in 107 patients who discontinued treatment for reasons other than progression was not reached at the data cut-off (**b**).

At the data cut-off, 61/108 patients (56.5%) who discontinued due to disease progression died, with a median post-progression OS of 12.2 months [95% CI: 9.2–22.0] (Fig. 4a).

Post-discontinuation median overall survival in 107 patients who discontinued the treatment for reasons other than progression and who were not lost at follow-up was not reached at data cut-off (95% CI 42.3-NA months, Fig. 4b).

## Discussion

To the best of our knowledge, this report is by far the largest series of patients with CLL and *TP53* aberrations treated with ibrutinib in first line. Keeping in mind the limitations of data registry analysis, we adopted TTD and OS as objective endpoints for our evaluation, and we were able to analyze 747 patients with a minimum potential FU of 16 months and a 26-month median follow-up in a nationwide study, with a 100% capture of patients who initiated the study drug. In previous registry or real-world analyses, a median follow-up of 17.5 and 24 months was reported by Mato et al. [16] and by Parikh et al. [26], respectively.

Overall, the estimated 36.6% discontinuation rate that we observed at 24 months in our study, along with the 82.6% survival rate and 76.6% PFS rate confirm that first-line treatment with ibrutinib is effective in high-risk CLL with *TP53* aberrations.

The efficacy of first-line ibrutinib in previous real-world analyses [12, 16, 25] and clinical trials [8, 9, 27] are summarized in Table 4. Noteworthy, our patient population had the highest median age (71 years), 43.6% of the patients had an ECOG-PS ≥ 1, 2.8% pre-existing severe heart disease and 9.1% renal impairment. The discontinuation rate in this large survey and in a previous study of 100 patients from 14 centers participating in the Italian CLL campus network [25] appears lower that in two U.S. studies which reported a 33% discontinuation rate at a median follow-up of 13.8 months in 111 patients [12] and a median TTD of 32.5 months in 254 patients with 17p- [16]. It is worth noting that in our country salvage treatment with other BTK inhibitors were not available outside of clinical trials during the study period and that venetoclax single agent was not available before 2018. This might have prompted a more stringent management of toxicities in order to maintain patients on an effective therapy in the absence of other options, thus explaining the relatively low incidence of discontinuations due to unacceptable toxicity in our study (37 patients corresponding to 9.3% of all discontinuation events and 17.2% of discontinuations except progression). In line with this policy is also the observation that the most common daily dose adopted over the entire treatment was 420 mg and that less than 15% of the overall population had an average daily dose less than 300 mg. The discontinuation rate would remain lower even if considering the possibility that minor side effects of ibrutinib could have contributed to discontinuation in 33 patients (8.3% of all discontinuation events and 15.3% of discontinuation events except progression) reported as due to patient/medical decision.

**Table 4:** Outcome of ibrutinib in first-line treatment of CLL with TP53 aberrations reported in real-world studies and in clinical trials.

N. of patients	Median age	Median follow-up (mos)	Discontinuation	Survival	ORR (%)	Type of study (reference)
747	71	26	36.6% at 24 mos.	82.6% at 24 mos	77.7	Present study
254	70	20.4	Median time to discontinuation 32.5 mos	Median 57.7 mos 88% at 12 mos	NR	RW [16]
111	68	13.8	33% overall	89% at 12 mos	82.3	RW [13]
100	71	24	32% at 24 mos.	92% at 24 months	79	RW [25]
89	65	49.8	54% overall	88% at 48 months	93	CT [9]
27	62	70	67% overall	79% at 6 years	96.2	CT [26]
34	63	78	50% overall	88% at 24 months	93.9	CT [8]

*NR* not reported, *RW* real world, *CT* clinical trial.

The estimated 82.6% survival probability at 24 months is in line with previous reports of real-world experiences and the higher survival probability observed in clinical trials can be reasonably accounted for by younger age and eligibility criteria of the patients enrolled in prospective studies [8, 9, 27].

Risk factors for a shorter TTD and OS at multivariable analysis (Table 3) were represented by age and ECOG-PS. A history of atrial fibrillation or pre-existing severe heart disease were associated with a higher probability to discontinue ibrutinib, whereas male sex was associated with higher risk of death. The latter observation might simply reflect the fact that life expectancy of men in Italy is 80.1 years, 4.7 years shorter than in women [28].

Interestingly, TTD and OS were consistent across all Italian regions and centers, suggesting that the experience of the prescribing centers did not likely play any role in the decision to discontinue the treatment by the physician. A similar observation was previously reported by the UK CLL forum in relapsed/refractory patients [11] and taken together these findings show that ibrutinib treatment is manageable in the real-world setting including both academic centers and community hospitals.

The prognostic value of del(17p) or TP53 aberrations alone or in combination is currently under investigation in CLL and previous studies in the era of chemoimmunotherapy reported mixed results, some studies revealing a worse outcome for multi-hit aberrations [29] and others finding similar outcome [30, 31]. In a recent analysis of patients enrolled in a clinical trial and treated with ibrutinib upfront or in the relapsed setting, Brieghel and coworkers [32] reported a significantly shorter OS and PFS in 42 patients with multi-hit *TP53* mutations compared with 9 patients with single-hit *TP53* aberration. Our analysis of a subgroup of previously untreated 496 patients (66.4% of the total population) showed that *TP53* aberrations in combination in 250 patients had no significant impact on TTD and OS as compared with *TP53* deletion in 112 patients or *TP53* mutations alone in 134 patients (Fig. 3). Because *TP53* haploinsufficiency may generate genetic instability and play a crucial role in tumorigenesis [33], it is reasonable to assume that bearing single or double *TP53* lesions have a minimal differential impact, if any, on the outcome with BTKi agents in this genetic subset of patients with CLL. Median post-progression survival of 12.2 months in 108 patients who discontinued because of disease progression shows that the overall outcome for this population remains poor, as previously noted in a real-world study and in a phase 2 study of a younger patient population which showed an 8-month and a 25-month median survival after progression, respectively [8, 24]. Interestingly, a prolonged survival was recorded in our patients who discontinued ibrutinib due to reasons other than progression, hinting at a better efficacy of second line treatment in this patient population.

Limitations for this study included lack of information on subsequent treatments for most of patients and on causes of mortality as well as a detailed collection of AEs in a real-world scenario. Further studies could also focus on the prognostic/predictive significance of del 17p burden, variant allele frequency of *TP53* mutations and specific *TP53* mutation in patients receiving ibrutinib as first-line treatment.

In conclusion these data show that ibrutinib is an effective first-line treatment for patients with CLL carrying *TP53* aberrations thanks to a nationwide real-world analysis of 747 patients treated at both large academic centers and community practice hospitals. Clinical characteristics at baseline (age, male sex, pre-existing comorbidities, ECOG-PS) may influence TTD or OS, whereas multi-hit or single-hit *TP53* aberrations had no impact on outcome in this high-risk population.

## Supplementary information

supplementary material

checklist

## Data Availability

Data were obtained from an administrative database and sharing is not applicable due to legal issues. No new data were created in this study.
