# Supplementary material for: Outcomes in patients with chronic lymphocytic leukemia and TP53 aberration who received first-line ibrutinib: a nationwide registry study from the Italian Medicines Agency
Source: Blood Cancer J. 2023 Jun 28;13(1):99. doi: 10.1038/s41408-023-00865-z (PMC10307816; doi:10.1038/s41408-023-00865-z)

**Supplementary materials**

1. Definition of time to treatment discontinuation and time to progression, death or unacceptable toxicity

Time to treatment discontinuation was defined as the time occurring between the date of the first administration of ibrutinib and the date of treatment discontinuation for any cause, including death or lost to follow-up, plus half the days of medication covered by one prescription. We considered subjects who did not attend the scheduled medical visit for the renewal of the prescription of ibrutinib for ≥ 30 days as “discontinued” even in the absence of the compilation of the “end of treatment form”. Since posology involves a daily intake of the drug and a prescription will allow for the dispensation of enough drug to cover 30, 60 or 90 days of therapy, we believe that adding 15, 30 or 45 days (half of the prescription period) will allow us to better model when discontinuation of the therapy occurred during the time frame covered by the last prescription, under the assumption that discontinuation events are randomly distributed across the 30, 60 or 90 days and average at day 15, 30 or 45. Time to progression, death or toxicity was defined as the time occurring between the date of the first administration of ibrutinib and the date of treatment discontinuation for progression, death or toxicity, plus half the days of medication covered by one prescription. The patients who discontinued ibrutinib for reasons other than progression, death or toxicity were censored at the time of discontinuation. The aforementioned strategy of adding half the prescription period also apply for time to progression, death or unacceptable toxicity. To calculate PDT for patients without an end of treatment form filled in the platform but with a date of death retrieved from the ANPR (see Methods section), death was considered the reason for treatment interruption only if it occurred between 120 days from the last recorded administration of the drug.

1. **Supplementary Table 1**. Patients’ characteristics associated with being lost to follow-up.

| Characteristics | HR | LCL | HCL |
| --- | --- | --- | --- |
| Gender M vs F | 0.791 | 0.503 | 1.244 |
| Age 65-69 vs Age <65 years | 3.186 | 1.343 | 7.555 |
| Age 70+ vs Age <65years | 2.903 | 1.535 | 5.489 |
| Ecog 1 vs 0 | 0.917 | 0.557 | 1.512 |
| Ecog 1 vs 2+ | 1.522 | 0.698 | 3.32 |
| Renal impairment no vs yes | 1.103 | 0.611 | 1.991 |
| Pre-existing severe heart disease yes vs no | 0.703 | 0.301 | 1.64 |
| History of atrial fibrillation yes vs no | 0.948 | 0.38 | 2.367 |
| days since diagnosis | 1.004 | 0.999 | 1.01 |
| RAI 2 vs RAI 0-1 stage | 2.093 | 1.114 | 3.936 |
| RAI 3 vs RAI 0-1 stage | 2.284 | 1.256 | 4.155 |
| RAI 4 vs RAI 0-1 stage | 4.868 | 2.345 | 10.106 |
| Bulky disease yes vs no | 0.553 | 0.346 | 0.883 |

1. **Supplementary Figure 1**. Average daily dose. The most common daily dose adopted over the entire treatment was 420 mg and that less than 15% of the overall population had an average daily dose less than 300 mg. 9 patients (1.2%) have an average daily dose greater than 450 milligrams that is probably due to erroneous imputation of dispensed milligrams by the pharmacists.


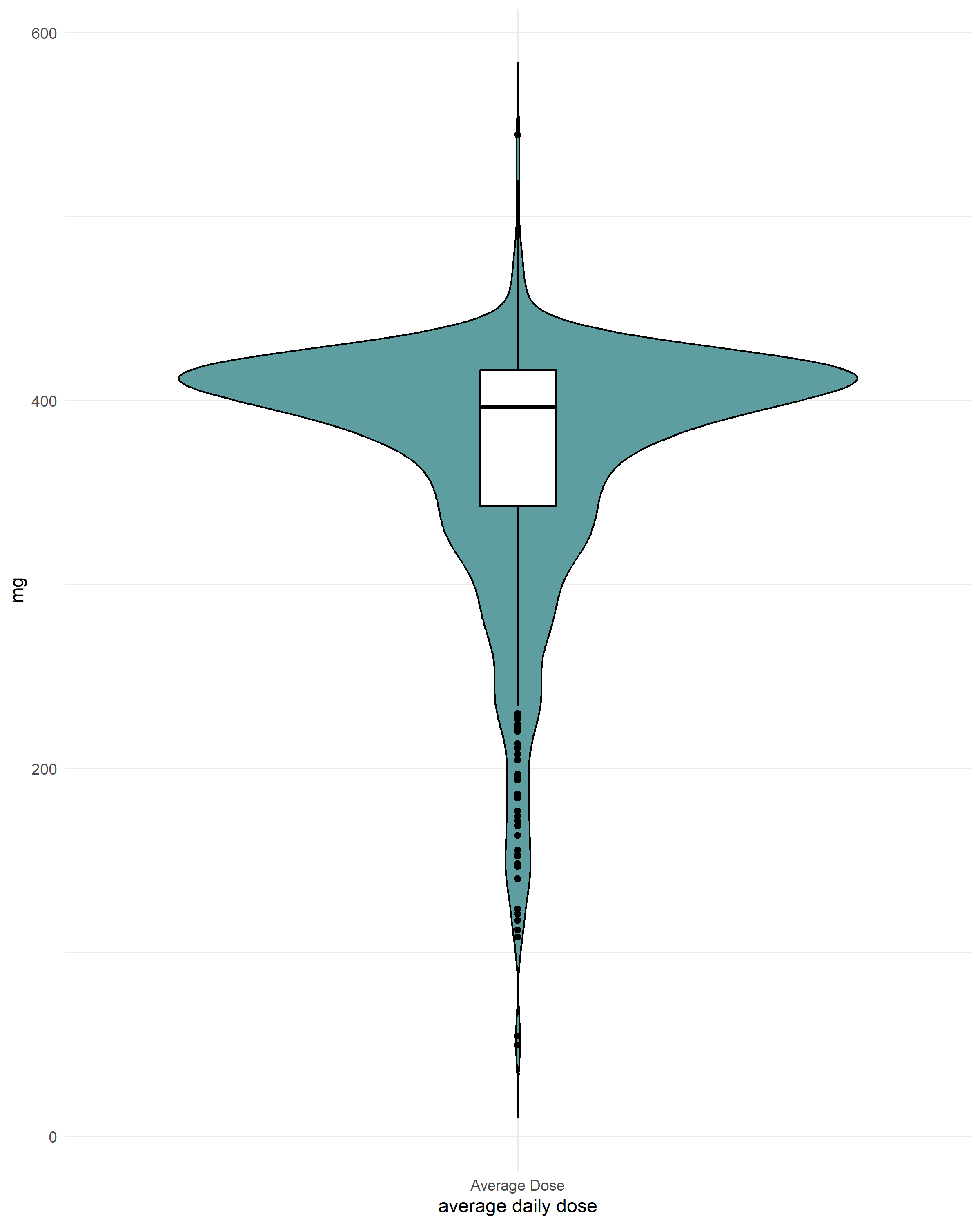


1. **Supplementary Table 2.** Starting dose of ibrutinib.

| Starting dose | N (%) |  |
| --- | --- | --- |
| 140 | 7 | 0.9% |
| 280 | 11 | 1.5% |
| 420 | 729 | 97.6% |
|  | 747 | 100.0% |

1. Multivariate analysis using Cox model with all baseline characteristics: TTD.

**Supplementary Table 3**: Hazard ratios from multivariate cox model on TTD including all baseline variables available for all patients.

| **Characteristics** | **HR** | **LCL** | **HCL** |
| --- | --- | --- | --- |
| Gender M vs F | 1.030 | 0.839 | 1.264 |
| Age 65-69 vs Age 64- | 1.139 | 0.819 | 1.586 |
| Age 70+ vs Age 64- | 1.802 | 1.389 | 2.337 |
| Ecog 1 vs 0 | 1.320 | 1.065 | 1.637 |
| Ecog 1 vs 2+ | 1.885 | 1.307 | 2.720 |
| Renal impairment no vs yes | 0.737 | 0.535 | 1.015 |
| Pre-existing severe heart disease yes vs no | 1.714 | 1.032 | 2.847 |
| History of atrial fibrillation yes vs no | 1.895 | 1.186 | 3.029 |
| days since diagnosis | 0.999 | 0.997 | 1.002 |
| RAI 2 vs RAI 0-1 | 0.895 | 0.679 | 1.178 |
| RAI 3 vs RAI 0-1 | 1.196 | 0.910 | 1.570 |
| RAI 4 vs RAI 0-1 | 0.955 | 0.705 | 1.293 |
| Bulky disease yes vs no | 1.028 | 0.816 | 1.294 |

Multivariate analysis using Cox model with all baseline characteristics: OS.

**Supplementary Table 4**: Hazard ratios from multivariate cox model on OS including all baseline variables available for all patients.

| **Characteristics** | **HR** | **LCL** | **HCL** |
| --- | --- | --- | --- |
| Gender M vs F | 1.352 | 0.992 | 1.842 |
| Age 65-69 vs Age 64- | 1.227 | 0.763 | 1.974 |
| Age 70+ vs Age 64- | 1.632 | 1.114 | 2.392 |
| Ecog 1 vs 0 | 1.391 | 1.015 | 1.907 |
| Ecog 1 vs 2+ | 2.534 | 1.573 | 4.082 |
| Renal impairment no vs yes | 0.713 | 0.454 | 1.118 |
| Pre-existing severe heart disease yes vs no | 1.901 | 0.938 | 3.854 |
| History of atrial fibrillation yes vs no | 0.912 | 0.414 | 2.008 |
| days since diagnosis | 1.000 | 0.996 | 1.004 |
| RAI 2 vs RAI 0-1 | 0.742 | 0.488 | 1.131 |
| RAI 3 vs RAI 0-1 | 1.119 | 0.749 | 1.67 |
| RAI 4 vs RAI 0-1 | 1.254 | 0.825 | 1.905 |
| Bulky disease yes vs no | 1.040 | 0.745 | 1.452 |

1. **Supplementary Figure 2**: Response rates at different time points


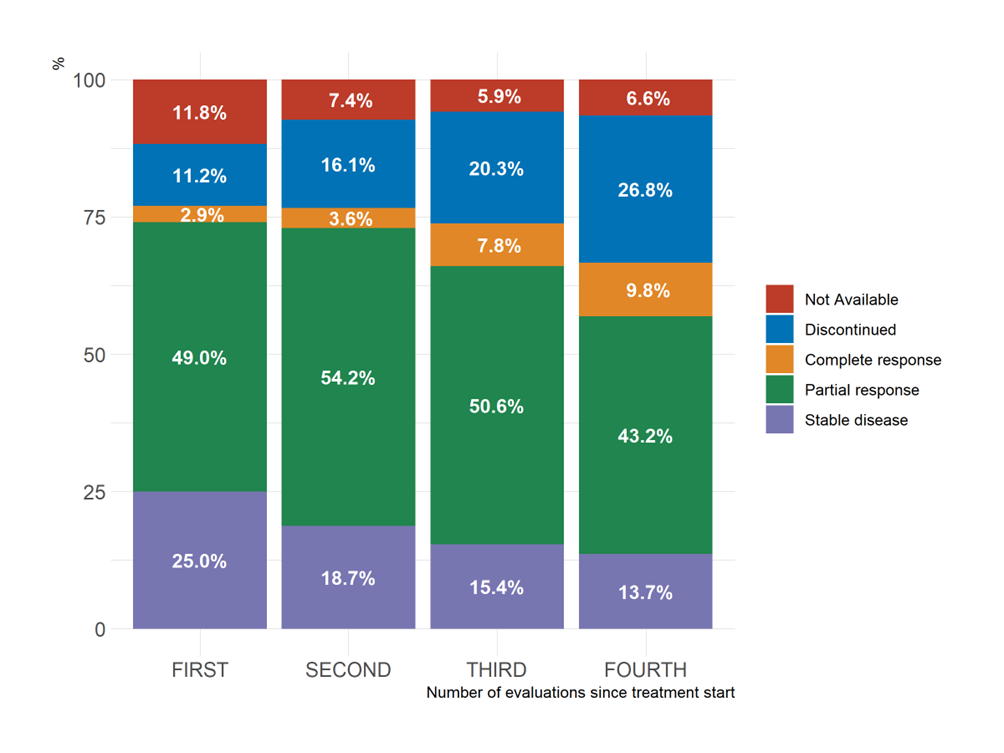


1. Accelerated failure time model.

In order to evaluate the association between TTD and baseline ECOG score, in the absence of proportional hazards assumptions, an AFT model has been estimated with an exponential parametric specification selected upon inspection of **Supplementary Figure 3:** Choice of the parametric specification for the AFT model.

The results observed are reported in the Supplementary Table 3 and confirmed a significant association between ECOG and TTD as discussed in the main text of the article.

**Supplementary Table 5**: Estimates of an exponential AFT model on TTD.

| **Characteristics** | **Time Rate** | **2.5 %** | **97.5 %** |
| --- | --- | --- | --- |
| (Intercept) | 2009.744 | 1378.679 | 2929.669 |
| AgeClass 65-69 vs 64- | 0.863 | 0.622 | 1.199 |
| AgeClass 70+ vs 64- | 0.554 | 0.429 | 0.716 |
| ECOGClass 1 vs 0 | 0.760 | 0.614 | 0.940 |
| ECOGClass 2+ vs 0 | 0.491 | 0.343 | 0.701 |
| Renal impairment yes vs no | 1.393 | 1.016 | 1.910 |
| Pre-existing severe heart disease yes vs no | 0.581 | 0.355 | 0.949 |
| History of atrial fibrillation yes vs no | 0.541 | 0.342 | 0.854 |

**Goodness of fit** for the AFT model is reported in **Supplementary Figure 4.**

1. Logistic regression comparing patients **with or without** a complete set of information on 17p and TP53.

**Supplementary Table 6:** Odds ratios and 95% confidence interval for baseline characteristics and time to treatment discontinuation of patients included in the subgroup analysis (with all information on TP53 aberration) vs patients not included (without all information on TP53 aberration)

| **Characteristics** | **OR** | **2.5 %** | **97.5 %** |
| --- | --- | --- | --- |
| (Intercept) | 2.44 | 0.88 | 6.98 |
| Gender M vs F | 0.99 | 0.72 | 1.37 |
| Age 65-69 vs Age 64- | 0.98 | 0.61 | 1.59 |
| Age 70+ vs Age 64- | 0.85 | 0.58 | 1.24 |
| RAI 1 vs RAI 0 | 0.90 | 0.42 | 1.87 |
| RAI 2 vs RAI 0 | 0.83 | 0.39 | 1.68 |
| RAI 3 vs RAI 0 | 0.77 | 0.36 | 1.57 |
| RAI 4 vs RAI 0 | 0.97 | 0.44 | 2.04 |
| Ecog 1 vs 0 | 0.99 | 0.71 | 1.38 |
| Ecog 1 vs 2+ | 1.00 | 0.54 | 1.91 |
| Pre-existing severe heart disease yes vs no | 0.73 | 0.30 | 1.88 |
| History of atrial fibrillation yes vs no | 0.80 | 0.35 | 1.91 |
| Bulky disease yes vs no | 0.99 | 0.70 | 1.40 |
| Renal impairment no vs yes | 0.98 | 0.56 | 1.67 |
| Days since diagnosis | 1.00 | 1.00 | 1.01 |
| Time to treatment discontinuation | 1.00 | 1.00 | 1.00 |

1. Standardized mean differences before and after weighting with gradient boosting (gbm) propensity score are shown in **Supplementary Figure 5**. All available baseline characteristics were eventually balanced among patients with single hit, del (17p) and TP53 separately and multi-hit aberrations as shown in the following love plots.


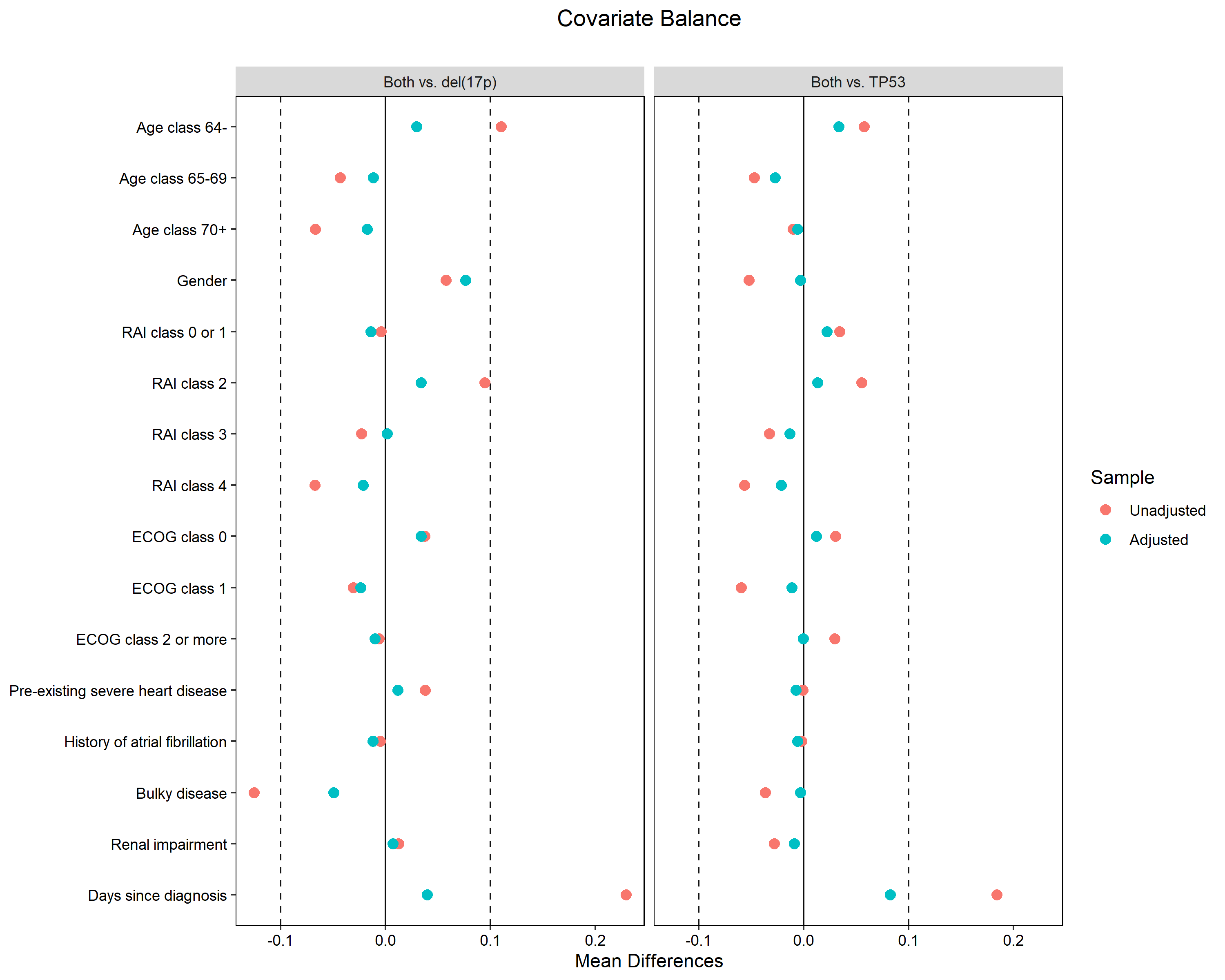

Supplement: Supplementary file 1 — supplementary material [file 41408_2023_865_MOESM1_ESM.docx]
